# Physical Activities Monitoring Using Wearable Acceleration Sensors Attached to the Body

**DOI:** 10.1371/journal.pone.0130851

**Published:** 2015-07-23

**Authors:** Muhammad Arif, Ahmed Kattan

**Affiliations:** Department of Computer Science, College of Computer and Information systems, Umm-Alqura University, Mecca, Saudi Arabia; Universidad Europea de Madrid, SPAIN

## Abstract

Monitoring physical activities by using wireless sensors is helpful for identifying postural orientation and movements in the real-life environment. A simple and robust method based on time domain features to identify the physical activities is proposed in this paper; it uses sensors placed on the subjects’ wrist, chest and ankle. A feature set based on time domain characteristics of the acceleration signal recorded by acceleration sensors is proposed for the classification of twelve physical activities. Nine subjects performed twelve different types of physical activities, including sitting, standing, walking, running, cycling, Nordic walking, ascending stairs, descending stairs, vacuum cleaning, ironing clothes and jumping rope, and lying down (resting state). Their ages were 27.2 ± 3.3 years and their body mass index (BMI) is 25.11 ± 2.6 Kg/m^2^. Classification results demonstrated a high validity showing precision (a positive predictive value) and recall (sensitivity) of more than 95% for all physical activities. The overall classification accuracy for a combined feature set of three sensors is 98%. The proposed framework can be used to monitor the physical activities of a subject that can be very useful for the health professional to assess the physical activity of healthy individuals as well as patients.

## Introduction

Physical activity monitoring and identifying human body positions are useful in many ways, including the development of personal weight control plans and the rehabilitation of patients in free living environments (environment where subject can move freely without any constraint of movement by the sensors on the body). Physical activity includes static postures, such as sitting, standing, etc., and dynamic motions include walking, running, jogging, etc. In smart environments (physical world where multiple sensors embedded in our daily routines to monitor different aspects of our living), the functional state of individuals can be monitored by inexpensive wireless sensors. Acceleration-based activity monitoring is successfully applied to a variety of populations, including healthy individuals and patients suffering from various diseases. Obesity is a disease/ condition that is becoming serious health problem in the world population due to physical inactivity [1]. It is becoming a major public health concern in developed, as well as developing, countries. Worldwide, according to the World Health Organization (WHO), obesity has nearly doubled since 1980 [2]. The WHO estimates that more than 10% of the world’s population is obese. To reduce the problem of obesity, preventative efforts include a proper diet and enhanced daily physical activities. It is reported in the literature that both diet and physical activity are important factors [3–5]. Many nutritionists and doctors monitor the physical activity of patients by self-completed questionnaires to assess the amount of physical activity [6]. A physical activity index that is based on the questionnaires are also proposed to assess different level of activity among the people [7]. Monitoring physical activity can also provide contextual knowledge for human-computer interaction applications [8] or provide information to the smart walking support systems about the intent of the user [9–11].

It is difficult and cumbersome to report the daily physical activity of individuals through the use of self-recorded reports. Hence, a great deal of research has been done in the past two decades on the use of wearable sensors for monitoring daily physical activities. The application of acceleration sensors for monitoring physical activity gained popularity in the last decade, as more accurate and cheaper sensors are available with the advancement of Micro-Electro-Mechanical Systems (MEMS) technology [12–15]. Acceleration-based monitoring systems can be integrated to provide more comprehensive intelligent in-home monitoring of physical activities [16–18]. Gubbi et al. [19] monitored the physical activities of stroke patients. Acceleration sensors are also used for other medical applications, including muscle activity classification [20].

Mathie et al. [21] presented a framework of a binary decision tree for the classification of various human movements, including rest, walking and falling by using a single tri-axial acceleration sensor that was placed at the waist. Sekine et al. [22] used a discrete wavelet transform to classify different types of walking, including walking on a level surface, walking upstairs and walking downstairs. Many systems investigated the classification of various physical activities by placing more than one sensor on the human body [23–25]. Mannini et al. [11] placed five acceleration sensors on a subject’s forearm, waist, wrist, thigh and ankle to classify seven types of physical activities by using different machine learning algorithms. Maurer et al. [24] placed multiple sensors on different body positions (wrist, belt, necklace, trouser pocket, shirt pocket and bag) to classify six types of activities and reported an accuracy of 80% to 92% for various sets of features and positions. Yang et al. [26] provided an interesting overview of accelerometer-based physical activity monitoring. Parkka et al. [17] used two acceleration sensors, one on the chest and another at the wrist to classify seven activities (lying down, row, ex-bike, sit/stand, run, Nordic walk and walk) and reported an average classification accuracy of 86% while using an automatic decision tree classifier. Attallah et al. [27] used wearable acceleration sensors on seven different body locations to recognize fourteen types of activities. Features including statistical, time and frequency domains resulted in a classification accuracy of about 90% by using a Bayesian classifier. A good review of human physical activity recognition using wearable sensors can be found in Lara and Labrador [28].

In this paper, twelve different types of activities (lying down, sitting, standing, walking, running, cycling, Nordic walking, ascending stairs, descending stairs, vacuum cleaning, ironing clothes and jumping rope) are classified using time domain features. A rotation forest classifier is used as classifier to classify the physical activities. Effective feature set reduction is achieved through correlation-based feature selection. In the end, the results reported in this paper are compared with published results to illustrate the effectiveness of the proposed framework.

## Materials and Methods

In this paper, the PAMAP2 dataset of physical activity monitoring [29] is used. This dataset is available free of cost from UCI Machine Learning Repository. The dataset can be downloaded from the website of UCI machine learning repository (https://archive.ics.uci.edu/ml/datasets/PAMAP2+Physical+Activity+Monitoring). Three Colibri wireless IMUs (Inertial Measurement Units) were placed on the subjects’ dominant-arm wrist and ankle, as well as chest. Nine subjects, including one woman and eight men, performed twelve different types of physical activities. Their age is 27.2 ± 3.3 years and BMI is 25.11 ± 2.6 Kg/m^2^. One subject was left-handed whereas rest of the subjects were right handed. All subjects were either students or employee of German Research Center for Artificial Intelligence (DFKI), Germany [30] and data was collected in 2011. The physical activities included lying down, sitting, standing, walking, running, cycling, Nordic walking, ascending stairs, descending stairs, vacuum cleaning, ironing clothes and jumping rope. Protocol of data collection was spanned on 45 minutes. The data collection protocol included the activities in the following manner; lying down for three minutes, sitting for three minutes, standing for three minutes, ironing the cloths for three minutes, a break of one minute, vacuum cleaning for three minutes, break of one minutes, ascending stairs for one minutes, break of two minutes, descending stairs for one minute, break for one minutes, ascending stairs again for one minute, descending stairs for one minutes, break for two minutes, normal walking for three minutes, break of one minute, Nordic walking for three minutes, break of one minutes, cycling for three minutes, break of one minute, running for three minutes, break of two minutes and rope jumping for two minutes. Futher details of the data collection is available in [30]. Each inertial measurement unit (IMU) contained a three dimensional (3D) acceleration sensor measuring up to ±16g with a resolution of 13 bits, a 3D gyroscope sensor, a 3D magnetometer sensor, and temperature and orientation sensors. In this study, we have only used the data of acceleration sensors from the whole dataset. Data from the acceleration sensors was sampled at 100Hz. A viliv S5 ultra-mobile personal computer (UMPC) manufactured by Viliv, South Korea with two universal serial bus (USB) dongles placed in companion bag is used to record the data. Labeling of the data was done on a GUI developed on UMPC.

### Time Domain Feature Extraction

A window of w_t_ seconds (*N* = *f*_*s*_ × *w*_*t*_ samples) is used to calculate the feature set for a particular activity. Here, *f*_*s*_ is the sampling frequency of the acceleration data. For each dimension of acceleration sensor, twelve different features in the time domain are extracted. Following is the detail of each time domain feature. Let *x* is the signal of window size w_t_ seconds having *N* samples or data points.

#### Mean Absolute Value (MAV)

The mean absolute value [31] is the summation of absolute values of all the data points of the signal in the window, divided by the window size *N*.

$$MAV=\frac{1}{N}\sum_{i=1}^{N}\left|x_{i}\right|$$

An extension of MAV is the windowed MAV in which data is multiplied with a particular window function *w*.

$$WMAV=\frac{1}{N}\sum_{i=1}^{N}w_{i}|x_{i}|$$

In this paper, we have used two types of window functions.

$$w_{1}(i)=\left\{ \begin{array}{cc} 1 & 0.25N\leq i\leq 0.75N\\ 0.5 & Otherwise \end{array}\right.$$

w2(i)={10.25N≤i≤0.75N4iN0.25N>i4(N-i)N0.75N<i

Hence, three features—MAV, WMAV1 (with window function *w*_1_) and WMAV2 (with window function *w*_2_)—are calculated from the data.

#### Harmonic Mean (HM)

The harmonic mean is defined as

$$HM=\frac{N}{\sum_{i=1}^{N}\frac{1}{x_{i}}}$$

#### Variance (VR)

The variance of data measures the spread of the data around the arithmetic mean. It is defined as

$$\sigma^{2}=\frac{\sum_{i=1}^{N}{\left(x_{i}-\mu \right)}^{2}}{N}$$

#### Root Mean Square (RMS)

The root mean square (RMS) is defined as

$$RMS=\sqrt{\frac{1}{N}\sum_{i=1}^{N}x_{i}^{2}}$$

#### Skewness (SK)

Skewness measures the asymmetry of the distribution of the data points of the acceleration data around the mean and is defined as

$$SK=\frac{{E(x-\mu )}^{3}}{\sigma^{3}}$$

where μ is the mean and σ is the standard deviation of the data.

#### Kurtosis (KT)

Kurtosis is a descriptor of the shape of the distribution of the data points of the acceleration data and is defined as

$$KT=\frac{{E(x-\mu )}^{4}}{\sigma^{4}}$$

Where μ is the mean and σ is the standard deviation of the data.

#### Cumulative Length (CL)

Cumulative length of the data is defined as follows:

$$CL=\sum_{i=1}^{N-1}\left|x_{i+1}-x_{i}\right|$$

#### Zero Crossing Rate (ZC)

The zero crossing rate [31] is the number of times the acceleration data crosses zero and changes its signs. The ZC is initialized as zero and is incremented when

$$\{x_{i}>0\;and\;x_{i+1}<0\}\;OR\;\{x_{i}<0\;and\;x_{i+1}>0\},1\leq i\leq N$$

#### Willison Amplitude (WA)

The Willison amplitude [32] is the number of times the absolute value of the difference of consecutive data points exceeds a pre-defined threshold ϵ.

$$WA=\sum_{i=1}^{N-1}f(|x_{i+1}-x_{i}|)$$

$$f(s)=\left\{ \begin{array}{cc} 1 & s>\epsilon \\ 0 & otherwise \end{array}\right.$$

#### Slope Sign Change (SSC)

The slope sign change [31] is the number of times the slope of the signal changes its sign. SSC is initialized as zero and incremented if

$$\{x_{i}>x_{i-1}\;and\;x_{i}>x_{i+1}\}\;OR\;\{x_{i}<x_{i-1}\;and\;x_{i}<x_{i+1}\},1\leq i\leq N$$

#### Simple Squared Integral

The simple squared integral calculates the energy of the signal.

$$SSI=\sum_{i=1}^{N}x_{i}^{2}$$

#### Correlation Coefficient (CC)

The correlation coefficient measures the correlation among the acceleration in *x*, *y* and *z* directions and among the different acceleration sensors.

### Classification of the physical activities

In this paper, we have compared the performance of three classifiers. The K nearest neighbor (KNN) [33] classifier is a widely used model-free classifier in which the classification of the data is decided according to the class labels of the neighboring instances. For a set of instances—DB and a query point ***q*** and parameter *K*—KNN returns a set of nearest neighbors *NN*_*q*_ such that

$$\forall i,j\;i\in {NN}_{q},j\in DB-{NN}_{q}:d(q,i)<d(q,j)$$

Here *d*(*q*,*i*) is any distance metric. The class of query point ***q*** will be decided by the majority class of *NN*_*q*_. The rotation forest is another type of ensemble classifier in which M decision trees are independently trained from different subsets of features [34,35]. For the rotation forest classifier, the user has to define the number of features in a subset, the number of classifiers in the ensemble, the extraction method and the base classifier.

The back-propagation neural network is well explained in Fausett [36]. It is a parallel information processing network that is quite well known in pattern classifications, due to its universal approximation capability for mapping functions.

### Feature Subset Selection

The feature subset selection is an important step to remove the redundant features from the feature set. It will improve the time and space complexity of the classification algorithms. In this paper, we have used the correlation-based feature selection (CFS) method [37,38] to select the feature subset. This method considers the prediction ability of each feature in the subset and redundancy of the feature with other features simultaneously. Hence, in the feature subset, a high correlation of the features to the classes and low inter-correlation among features is desirable. The linear correlation coefficient is used to determine out the correlation among the feature subset.

Different search methods can be used to find the feature subset in the CFS technique. We have used a scatter search [39], which uses the diversification generation method to generate diverse subsets. We then passed them through an improvement method, which is usually a local search in the initial phase. A reference set is built on the initial sets and subsets are generated from the reference set. The main loop of the scatter search consists of subset generation, solution combination, improvement method and reference set update method. This loop is terminated based on the stopping condition that uses a threshold value [40]. Lopez et al. [39] developed three scatter search base algorithms: a sequential scatter search with a greedy combination (SS-GC), a sequential scatter search with a reduced greedy combination (SS-RGS) and a parallel scatter search. In this paper, the sequential scatter search with a reduced greedy combination is used to select the optimal feature subset.

### Analysis of Results

Acceleration-based physical monitoring algorithms can be validated in identifying different postures and movements using precision, recall and F-measure. The precision or positive predictive value (PPV) is defined as the proportion of instances that belongs to a class (TP: True Positive) by the total instances, including TP and FP (False Positive) classified by the classifier as belong to this particular class.

$$Precision=\frac{TP}{TP+FP}$$

The recall or sensitivity is defined as the proportion of instances classified in one class by the total instances belonging to that class. The total number of instances of a class includes TP and FN (False Negative).

$$Recall=\frac{TP}{TP+FN}$$

The F-measure is the combination of precision and recall and is defined as

$$F-measure=\frac{2\times Precision\times Recall}{Precision+Recall}$$

### Results and Discussion

As discussed in Section of Materials and methods, a sliding window *w*_*t*_ of 5 seconds is applied to the acceleration data in *x*, *y* and *z* directions of the three sensors and the features are extracted from this window to identify the activity as being one of twelve possible options. The sampling frequency to record the acceleration data is 100Hz. An overlap of one second is considered for sliding the window on the acceleration data. Features are calculated on the window of 5 seconds and three features sets (FS1, FS2, FS3) of 18664 instances are obtained for three sensors placed on the chest, wrist and ankle, respectively. Table 1 shows the distribution of instances of the activities in the feature sets.

**Table 1:** Number of instances per activity.

Physical Activity	Number of Instances
A1: Lying	1857
A2: Sitting	1783
A3: Standing	1832
A4: Walking	2321
A5: Running	931
A6: Cycling	1585
A7: Nordic walking	1821
A8: Ascending stairs	1102
A9: Descending stairs	981
A10: Vacuum cleaning	1685
A11: Ironing	2317
A12: Rope jumping	449
**Total**	**18664**

Features are calculated on MATLAB (MathWorks Natick, MA, United States of America) for all the activities. The whole feature set is randomly divided into two sets. The training set includes 70% of the instances from the features set and is used for training the classifiers, whereas the testing set includes 30% of the instances from the feature set and is used for testing the classification performance of the classifiers. The classification results of all of the physical activities using three 3D acceleration sensors separately and a combination of all three sensors are reported in the next sections.

### Classification results using acceleration sensor data placed on the wrist

In this section, the classification results of all physical activities using the acceleration sensor placed on the wrist are described. The time domain features set (FS1) is comprised of 39 features that were extracted from the acceleration data in the *x*, *y* and *z* directions. The classification results of three classifiers, the KNN, rotation forest and the back-propagation neural network, are reported in Table 2. All classifiers are trained on the training set and the classification results in Table 2 are obtained by using the testing set. Precision, recall and F-measure are reported for every classifier. The overall classification accuracy for KNN, rotation forest and neural network are 70%, 94% and 89%, respectively. The KNN classifier performed poorly in distinguishing the activities of walking, running, Nordic walking and rope jumping. Although PPV or the precision of all these activities are quite high, but poor sensitivity produced low F-measure values. Hence, it is evident that the ratio of true positive (TP) is high, but there are a large number of false negatives (FN), thus resulting in low values of sensitivity. Classification results of neural network classifiers are the second best, and both precision and recall of all of the activities are above 0.7. The rotation forest classifier was able to classify all of the activities with reasonable accuracy and the F-measure for all physical activities is almost over 90%.

**Table 2:** Classification results of three classifiers using the acceleration sensor placed at dominant wrist.

	KNN (K = 3)			Rotation Forest			Neural Network		
Activity	Precision	Recall	F-measure	Precision	Recall	F-measure	Precision	Recall	F-measure
**A1**	0.927	0.933	0.930	0.974	0.940	0.957	0.890	0.898	0.894
**A2**	0.910	0.834	0.870	0.922	0.895	0.908	0.857	0.804	0.830
**A3**	0.892	0.809	0.848	0.855	0.924	0.888	0.756	0.862	0.806
**A4**	0.875	0.088	0.160	0.984	0.954	0.969	0.957	0.897	0.926
**A5**	0.154	0.971	0.266	0.996	0.957	0.976	0.964	0.953	0.958
**A6**	0.984	0.888	0.933	0.987	0.983	0.985	0.991	0.966	0.979
**A7**	0.994	0.317	0.480	0.989	0.977	0.983	0.987	0.979	0.983
**A8**	0.904	0.864	0.884	0.907	0.896	0.902	0.786	0.855	0.819
**A9**	0.945	0.820	0.878	0.954	0.852	0.900	0.961	0.703	0.812
**A10**	0.983	0.828	0.899	0.961	0.939	0.950	0.937	0.929	0.933
**A11**	0.898	0.811	0.852	0.845	0.962	0.900	0.830	0.928	0.876
**A12**	0.931	0.190	0.316	0.963	0.915	0.939	0.903	0.915	0.909
**Average**	**0.890**	**0.688**	**0.708**	**0.941**	**0.939**	**0.939**	**0.900**	**0.895**	**0.896**

The best classification results are obtained by using a rotation forest classifier. The precision of standing and ironing activities were low (0.85 and 0.845, respectively), whereas the recall of sitting, ascending and descending stairs were low (less than 90%).

The confusion matrix of all activities using a rotation forest classifier is given in Table 3. Some instances of sitting and standing activities are confused with each other and also with the ironing activity. Similarly, some instances of walking were confused with ascending stairs. Interestingly, a few instances of ascending and descending stairs are confused with standing and ironing activities.

**Table 3:** Confusion matrix for rotation forest classifier (Acceleration sensor placed at wrist).

	A1	A2	A3	A4	A5	A6	A7	A8	A9	A10	A11	A12
**A1**	561	8	6	0	0	1	1	1	0	7	12	0
**A2**	6	475	24	0	0	0	1	2	1	4	18	0
**A3**	4	17	489	0	0	1	0	1	2	1	14	0
**A4**	0	1	2	682	0	0	0	15	3	0	8	4
**A5**	2	0	3	1	265	0	3	2	0	0	1	0
**A6**	1	0	0	0	0	464	0	0	2	0	5	0
**A7**	0	2	0	3	0	0	546	0	0	3	4	1
**A8**	1	2	10	3	1	1	0	284	4	0	11	0
**A9**	0	1	18	3	0	1	0	7	270	0	17	0
**A10**	0	0	6	0	0	1	0	1	1	449	20	0
**A11**	1	7	12	0	0	1	1	0	0	3	640	0
**A12**	0	2	2	1	0	0	0	0	0	0	7	130

### Classification results using acceleration sensor data placed on the chest

The time domain features set (FS2) was extracted from the acceleration data of the sensor that was placed on the chest during all physical activities in a similar fashion as described in Section of Materials and methods. The classification results are tabulated in Table 4 for the same three classifiers on the testing set. The KNN classifier performed poorly on the feature set of the chest sensor. The overall classification accuracy is 70% for the KNN classifier. Either the precision or recall of some activities is poor, thereby resulting in the overall poor value of the F-measure. The precision of the running activity is found to be very low. The overall classification accuracy of the neural network classifier is found to be 86%, with F-measure of more than 0.80 for most of the activities. The rotation forest classifier was performed the best among these three classifiers and the overall accuracy is found to be 93.8% with an F-measure of all the physical activities more than 0.90.

**Table 4:** Classification results of three classifiers using the acceleration sensor placed on the chest.

	KNN (K = 3)			Rotation Forest			Neural Network		
Activity	Precision	Recall	F-measure	Precision	Recall	F-measure	Precision	Recall	F-measure
A1	0.988	0.930	0.958	0.985	0.977	0.981	0.976	0.938	0.956
A2	0.873	0.802	0.836	0.917	0.893	0.905	0.699	0.791	0.742
A3	0.839	0.758	0.796	0.838	0.930	0.882	0.729	0.665	0.696
A4	0.916	0.476	0.626	0.952	0.966	0.959	0.855	0.913	0.883
A5	0.221	0.968	0.359	0.996	0.957	0.976	0.974	0.957	0.965
A6	0.981	0.860	0.916	0.958	0.972	0.965	0.979	0.871	0.922
A7	0.979	0.658	0.787	0.974	0.925	0.949	0.886	0.807	0.845
A8	0.943	0.883	0.912	0.942	0.927	0.935	0.829	0.874	0.851
A9	0.985	0.833	0.903	0.976	0.883	0.927	0.950	0.845	0.895
A10	0.893	0.822	0.856	0.928	0.923	0.925	0.858	0.900	0.878
A11	0.881	0.798	0.838	0.902	0.938	0.920	0.824	0.904	0.862
A12	1.000	0.613	0.760	0.993	0.944	0.968	0.977	0.894	0.934
**Average**	**0.891**	**0.771**	**0.805**	**0.940**	**0.938**	**0.939**	**0.864**	**0.860**	**0.861**

The confusion matrix for the rotation forest classifier in Table 5 shows that some instances of sitting and standing activities are confused with each other. A similar trend was also observed for the sensor placed at the wrist (see Table 3). Ten instances of walking were confused with Nordic walking and, similarly, 26 instances of Nordic walking were confused with the walking activity. Some instances of vacuum cleaning and ironing are confused with each other as well. For 20 instances, the ironing activity was confused with the standing activity. Looking at the confusion matrix, it is obvious that similar activities or having similar postures while performing the activities were confused with each other. But most of the instances of all the activities are classified correctly.

**Table 5:** Confusion matrix for rotation forest classifier (Acceleration sensor placed on chest).

	A1	A2	A3	A4	A5	A6	A7	A8	A9	A10	A11	A12
**A1**	583	6	7	0	0	0	0	0	0	1	0	0
**A2**	3	474	35	0	0	1	0	1	0	4	13	0
**A3**	1	23	492	0	0	0	0	1	0	0	12	0
**A4**	0	0	4	691	0	6	10	1	0	2	1	0
**A5**	0	1	1	0	265	0	1	2	2	2	2	1
**A6**	2	0	0	0	0	459	0	0	0	9	2	0
**A7**	0	1	0	26	0	1	517	3	3	1	7	0
**A8**	0	4	9	2	0	5	1	294	1	0	1	0
**A9**	1	1	16	5	1	0	2	7	280	0	4	0
**A10**	1	0	2	2	0	5	0	3	0	441	24	0
**A11**	1	5	20	0	0	0	0	0	0	15	624	0
**A12**	0	2	1	0	0	2	0	0	1	0	2	134

### Classification results using acceleration sensor data placed on the ankle

Time domain features (FS3) are extracted from the sensor placed on the dominant ankle and the classification results revealed that the rotation forest classifier outperformed the other two classifiers, as shown in Table 6. The KNN classifier failed to distinguish among various physical activities, including walking, running, cycling, Nordic walking and jumping rope. The main reason of this low performance of KNN is either low precision or low recall of different activities. The neural network classifier showed that the moderate classification performance with the F-measure of most of the activities is above 0.80.

**Table 6:** Classification results of three classifiers using the acceleration sensor placed on the dominant ankle.

	KNN (K = 3)			Rotation Forest			Neural Network		
Activity	Precision	Recall	F-measure	Precision	Recall	F-measure	Precision	Recall	F-measure
A1	0.986	0.935	0.960	0.995	0.958	0.976	0.981	0.935	0.957
A2	0.870	0.821	0.845	0.963	0.883	0.921	0.786	0.734	0.759
A3	0.777	0.533	0.632	0.838	0.822	0.830	0.635	0.628	0.631
A4	0.895	0.071	0.132	0.968	0.958	0.963	0.925	0.829	0.875
A5	0.103	0.978	0.187	0.981	0.957	0.969	0.985	0.957	0.971
A6	0.962	0.216	0.353	0.949	0.981	0.965	0.887	0.932	0.909
A7	0.976	0.073	0.136	0.962	0.946	0.954	0.800	0.896	0.846
A8	0.967	0.820	0.887	0.951	0.918	0.934	0.950	0.909	0.929
A9	0.975	0.729	0.834	0.965	0.874	0.917	0.962	0.804	0.876
A10	0.867	0.586	0.699	0.853	0.921	0.885	0.778	0.843	0.809
A11	0.815	0.605	0.694	0.787	0.899	0.839	0.719	0.821	0.767
A12	1.000	0.162	0.279	1.000	0.937	0.967	0.978	0.923	0.949
**Average**	**0.865**	**0.525**	**0.558**	**0.925**	**0.921**	**0.922**	**0.846**	**0.840**	**0.841**

The rotation forest classifier is able to distinguish all physical activities with high accuracy. The F-measure is below 0.9 for the activities of standing, vacuum cleaning and ironing.

The confusion matrix for the rotation forest classifier (Table 7) revealed that 74 instances of standing were misclassified as ironing, which seems obvious since both activities are somewhat similar and the sensor is placed at ankle.

**Table 7:** Confusion matrix for rotation forest classifier (Acceleration sensor placed on ankle).

	A1	A2	A3	A4	A5	A6	A7	A8	A9	A10	A11	A12
**A1**	572	1	12	0	0	5	0	1	0	1	5	0
**A2**	1	469	27	0	0	0	0	1	1	12	20	0
**A3**	1	8	435	0	0	0	0	0	1	10	74	0
**A4**	0	0	1	685	0	3	18	2	3	2	1	0
**A5**	0	0	1	0	265	2	0	3	0	2	4	0
**A6**	0	0	0	0	0	463	0	0	1	4	4	0
**A7**	0	1	0	18	0	2	529	0	2	1	6	0
**A8**	0	1	5	2	0	2	2	291	0	4	10	0
**A9**	0	1	7	2	3	6	0	5	277	7	9	0
**A10**	0	1	1	1	0	5	1	3	0	440	26	0
**A11**	1	4	29	0	0	0	0	0	1	32	598	0
**A12**	0	1	1	0	2	0	0	0	1	1	3	133

The activities of vacuum cleaning and ironing have some instances that were confused. The reason may be due to some episodes of standing while vacuum cleaning and the acceleration recorded from the sensor placed on ankle failed to differentiate the activities. It is also evident from the table that the ironing activity was not only confused with the vacuum cleaning activity as well as the standing activity.

### Classification results using all three acceleration sensor’s data

Different physical activities involve the motion of different body parts, depending on the type of activity. For example, the activities of standing, vacuum cleaning and ironing involved more motion of the hand than the whole body. Hence, it is easier to classify different physical activities correctly by integrating the features extracted from the different sensors. In the previous sections, the sitting and standing activities had low classification accuracies in compared to other activities in all three locations of sensors. Similarly, ascending and descending stairs are poorly classified by the sensors located on the chest and wrist. Vacuum cleaning and ironing activities have low classification accuracy when a sensor is placed on the ankle.

Therefore, in this section, features extracted from all three sensors are integrated and all three classifiers are applied to discriminate the set of physical activities. The total features set for all three sensors (FS4 = FS1 ∪ FS2 ∪ FS3) were 117. The classification results of all three classifiers are summarized in Table 8. It can be observed from the table that all classifiers performed equally well on most of the physical activities.

**Table 8:** Classification results of three classifiers using all acceleration sensors.

	KNN (K = 3)			Rotation Forest			Neural Network		
Activity	Precision	Recall	F-measure	Precision	Recall	F-measure	Precision	Recall	F-measure
A1	0.993	0.990	0.992	0.995	0.993	0.994	0.995	0.988	0.992
A2	0.968	0.972	0.970	0.979	0.979	0.979	0.968	0.966	0.967
A3	0.942	0.989	0.965	0.956	0.983	0.969	0.941	0.974	0.957
A4	0.990	0.993	0.992	0.999	0.990	0.994	0.988	0.996	0.992
A5	0.996	0.975	0.985	1.000	0.971	0.985	0.985	0.975	0.980
A6	1.000	0.996	0.998	0.998	0.994	0.996	0.994	0.994	0.994
A7	0.996	0.989	0.993	0.993	0.987	0.990	0.988	0.989	0.988
A8	0.981	0.972	0.976	0.969	0.972	0.970	0.962	0.946	0.954
A9	0.983	0.934	0.958	0.964	0.934	0.949	0.983	0.912	0.946
A10	0.987	0.983	0.985	0.987	0.981	0.984	0.989	0.977	0.983
A11	0.973	0.994	0.984	0.959	0.994	0.976	0.967	0.982	0.975
A12	1.000	0.937	0.967	1.000	0.951	0.975	0.906	0.951	0.928
**Average**	**0.983**	**0.982**	**0.982**	**0.983**	**0.982**	**0.982**	**0.976**	**0.976**	**0.976**

Precision and recall of all the physical activities are more than 0.95 in all classifiers. The rotation forest classifier and KNN classifiers are fractionally better than neural network classifier.

The confusion matrix for the rotation forest classifier (Table 9) revealed that all physical activities are classified correctly and very few of the instances are misclassified. Hence, it becomes evident that placing acceleration sensors on various body parts can distinguish a larger set of physical activities. Very few instances of sitting and standing activities are confused with the ironing activity (9 and 6, respectively).

**Table 9:** Confusion matrix for rotation forest classifier (All Acceleration sensors).

	A1	A2	A3	A4	A5	A6	A7	A8	A9	A10	A11	A12
A1	591	1	1	0	0	0	0	0	0	2	2	0
A2	0	516	3	0	0	0	0	0	0	3	9	0
A3	0	7	515	0	0	0	0	0	1	0	6	0
A4	0	0	0	707	0	0	0	2	5	0	1	0
A5	0	1	2	1	266	0	1	3	1	1	1	0
A6	0	0	0	0	0	471	0	0	0	1	0	0
A7	0	2	1	1	0	0	551	0	0	1	3	0
A8	0	1	8	0	0	0	1	303	0	0	4	0
A9	0	2	9	2	0	0	1	8	287	3	5	0
A10	0	1	2	2	0	1	0	1	0	466	5	0
A11	0	1	6	0	0	0	0	0	0	5	653	0
A12	0	1	0	0	0	2	0	2	1	0	3	133

It is assumed that some attributes may be redundant in the feature set FS4. Hence, the correlation-based feature selection (CFS) is used to remove the redundant and irrelevant features and to reduce the feature set. A reduced scatter search (RSS) is used to search for the best feature subset. The reduced scatter search method generated the feature subset (FS5) comprising 46 features. The classification results of all three classifiers are reported in Table 10 for the features set FS5. It can be observed that, on the cost of fractional loss of classification accuracy, the number of features is reduced dramatically to 46, showing about a 60% reduction in the size of the feature set.

**Table 10:** Classification results of three classifiers on FS5 features set.

	KNN (K = 3)			Rotation Forest			Neural Network		
Activity	Precision	Recall	F-measure	Precision	Recall	F-measure	Precision	Recall	F-measure
A1	0.995	0.992	0.993	0.997	0.992	0.994	0.990	0.987	0.988
A2	0.975	0.970	0.973	0.973	0.959	0.966	0.916	0.964	0.939
A3	0.949	0.985	0.967	0.937	0.983	0.959	0.910	0.932	0.921
A4	0.975	0.993	0.984	0.999	0.989	0.994	0.996	0.993	0.994
A5	1.000	0.975	0.987	0.993	0.971	0.982	0.971	0.975	0.973
A6	1.000	0.998	0.999	0.998	0.994	0.996	0.987	0.987	0.987
A7	0.996	0.986	0.991	0.991	0.989	0.990	0.991	0.984	0.987
A8	0.974	0.959	0.967	0.959	0.956	0.957	0.967	0.921	0.943
A9	0.966	0.909	0.937	0.960	0.918	0.939	0.970	0.921	0.945
A10	0.983	0.981	0.982	0.973	0.975	0.974	0.969	0.973	0.971
A11	0.964	0.994	0.979	0.956	0.991	0.973	0.964	0.965	0.965
A12	1.000	0.930	0.964	1.000	0.937	0.967	0.971	0.930	0.950
**Average**	**0.980**	**0.979**	**0.979**	**0.977**	**0.977**	**0.977**	**0.967**	**0.967**	**0.967**

The performance of the KNN classifier (F-measure 0.97) is slightly better than the rotation forest classifier (F-measure 0.977). The confusion matrix for the KNN classifier is reported in Table 11, which shows excellent classification accuracies for all types of physical activities.

**Table 11:** Confusion matrix for KNN classifier on features set FS5.

	A1	A2	A3	A4	A5	A6	A7	A8	A9	A10	A11	A12
A1	592	3	0	0	0	0	0	0	0	1	1	0
A2	1	515	4	0	0	0	0	0	2	2	7	0
A3	1	4	521	0	0	0	0	1	0	0	2	0
A4	1	0	2	710	0	0	0	0	2	0	0	0
A5	0	0	2	2	270	0	0	2	0	1	0	0
A6	0	0	0	0	0	471	0	0	0	1	0	0
A7	0	0	1	2	0	0	551	0	0	1	4	0
A8	0	1	4	2	0	0	0	304	3	1	2	0
A9	0	1	12	8	0	0	0	4	288	0	4	0
A10	0	2	2	1	0	0	2	0	0	469	2	0
A11	0	0	1	1	0	0	0	0	1	1	661	0
A12	0	2	0	2	0	0	0	1	2	0	3	132

Table 12 summarizes some of the published literature that relates to our paper on different datasets. This comparison is not fair in the sense that the results reported in the table are not on the same dataset. But in our view, purpose of the physical activity monitoring is to use minimum set of sensors placed on the body and to classify maximum number of physical activities with optimal features set (optimal in the sense that number of features are minimum and easy to compute). Parkka et al. [17] used time and frequency domain features to classify seven activities (lying down, rowing, riding bike, standing, running, walking and Nordic walking) and achieved a 94% overall accuracy. Bao et al. [23] reported excellent results on 20 ambulation and daily activities by using five sensors. They have used time and frequency domain features and obtained 95% accuracy for ambulation activities only. Overall accuracy was reported as 84%. Mantyjarvi et al. [41] used wavelet-based features to distinguish four activities (start/stop, level walk, upstairs and downstairs) using wavelet-based features. Zhu et al. [42] collected acceleration data by putting two sensors on wrist and waist and identified 12 different types of activities using hidden markov models with 90% classification accuracy. McGlynn et al. [43] proposed an ensemble classifier based on dynamic time warping and achieved 84% of classification accuracy on five types of activities using three sensors placed on thigh, hip and wrist.

**Table 12:** Comparison of performance with the reported results.

Reference	Number of Activities	Number of Sensors	Classification Accuracy
[17]	9	3 (wrist, chest, ankle)	86%
[23]	20	5 (forearm, wrist, waist, ear, thigh, ankle)	84%
[44]	5	1 (chest)	93%
[45]		2 (thigh)	92% to 95%
[41]	4	6 (3 left hip, 3 right hip)	83% to 90%
[42]	12	2 (wrist, waist)	90%
[43]	5	3 (thigh, hip, wrist)	84%
[46]	5 grouped activities	2 (wrist, hip)	88%
[47]	14	6 (thigh, chest, hip, both wrists, weapon)	90%
[48]	4	2 (thigh, trunk)	90%
[49]	8	3 (waist, thigh, ankle)	94%
**This paper**	**12**	**3 (chest, wrist, ankle)**	**98%**

Lee et al. [46] proposed a novel implementation of a semi-Markov conditional random field classifier. Physical activities of 24 types were grouped together into five groups (dinner, commuting, lunch, office work and routines) and obtained an average classification accuracy of 88%. A threshold-based posture monitoring system is proposed in [48] for a single elderly subject in the rehabilitation center. For every posture, authors defined some threshold values and a decision tree is used to identify different types of postures and the detection accuracies are in the order of 90%.

In our paper, we have distinguished twelve different types of physical activities with simple time domain features and obtained an overall classification accuracy of 98% by fusing the features of acceleration sensors placed on three body locations. All three classifiers have shown excellent accuracy on the fused feature set FS4. By our feature set, we were able to distinguish some difficult types of activities as well, such as walking downstairs and walking upstairs, which are reported to be confused in the literature [50,51]. The size of the feature set is important while implementing the physical activity monitoring system in real-life scenarios working wirelessly and using the battery power. On the account of fractional loss of classification accuracy, we have found a reduced feature set comprising of only 40% of the features (FS5) as compared to the full feature set (FS4).

## Conclusions

In this paper, acceleration sensors are placed on three body locations to identify twelve different types of physical activities. Time domain features were extracted from the acceleration sensors and the performance of three classifiers are compared for each location of sensor separately and combination with all three sensors. It is evident from the results that all twelve types of physical activities can be distinguished successfully if the feature set of the three sensors is used for the classification. The rotation forest classifier is found to be the best among the three classifiers in all settings. An average classification accuracy of 98% is obtained by rotation forest classifier. Furthermore, the combined feature set (FS4) is reduced by removing the redundant features using the correlation based feature selection method. Average classification accuracy on the reduced feature set is found to be 97.9% and 97.7% for the KNN and rotation forest classifiers, respectively. Performance of the proposed framework is compared with the published literature in Table 12 and it is evident from the table that our proposed framework performed better than the published results. Our proposed method can be used to build a wearable physical monitoring system that can increase the user’s awareness about his/her physical activity profile and promote a healthy life style for the people. In our future research, we will focus on finding optimal set of sensors placed on the appropriate locations on the body or stitched to the tight cloths worn by the person to monitor larger set of physical states or activities.
